# Predictors of suicidal ideation in Italian veterinarians

**DOI:** 10.1038/s41598-024-68330-w

**Published:** 2024-07-30

**Authors:** Giorgia Varallo, Andrea Zagaria, Valentina Baldini, Alessandro Schianchi, Marta Brscic, Matteo Panero, Christian Franceschini, Adriano Schimmenti, Alessandro Musetti

**Affiliations:** 1 Department of Biomedical, Metabolic and Neural Sciences, University of Modena and Reggio Emilia, Modena, Italy; 2 Department of Psychology, Sapienza University of Rome, Rome, Italy; 3 Department of Biomedical and Neuromotor Sciences, University of Bologna, Bologna, Italy; 4 Fornovo di Taro, Parma, Italy; 5 Department of Animal Medicine, Production and Health (MAPS), University of Padova, Viale dell’Università 16, 35020 Legnaro, PD Italy; 6 Department of Neuroscience “Rita Levi Montalcini”, Eating Disorders Center, University of Turin, Turin, Italy; 7 Department of Medicine and Surgery, University of Parma, Parma, Italy; 8 Faculty of Human and Social Sciences, UKE - Kore University of Enna, Enna, Italy; 9 Department of Humanities, Social Sciences and Cultural Industries, University of Parma, Parma, Italy

**Keywords:** Health care, Medical research

## Abstract

Suicide represents a significant problem for healthcare professionals such as veterinarians. Previous studies showed that contextual and individual risk factors can contribute to suicidality among veterinarians. In the present study, self-report measures on exposure to animal euthanasia, substance abuse, reflective functioning, and suicidal ideation were administered to 1556 Italian veterinarians aged 24–74 years old. Structural equation modelling revealed that failures in reflective functioning and substance abuse were associated with suicidal ideation. Prevention programs focusing on improving reflective functioning and decreasing substance abuse might reduce suicide risk among veterinarians.

## Introduction

The phenomenon of suicide is a matter of profound concern on a global scale, with approximately one million individuals dying by suicide annually^1^. The ramifications of suicide are not limited to the loss of human lives; they extend to substantial social and economic burdens^2^. Suicidality encompasses a spectrum of thoughts and behaviors ranging from suicidal ideation (i.e., suicidal thoughts, desires, and preoccupations) to suicide attempts to complete suicide^3^. A significant body of research has been dedicated to the epidemiology of suicide, revealing alarming trends within specific occupational categories. This is particularly evident among individuals engaged in professions related to human and animal care, which includes, but is not limited to, physicians, nurses, and veterinarians^4,5^. Veterinarians exhibit an elevated vulnerability to suicide, with several studies consistently reporting a higher prevalence of suicidal ideation and fatal suicide attempt risk that is up to fourfold greater than that of the general population^6–8^. Notably, suicidal ideation is a strong and consistent predictor of suicide attempts, underscoring the critical importance of identifying and addressing risk factors for suicidal ideation at an earlier stage, in order to prevent the progression to more severe suicidal behaviors^9,10^.

### Contextual risk factors

The principal risks associated with suicidal ideation can be categorized into two overarching domains: contextual and individual risk factors. Contextual risk factors, as the term implies, pertain to external environmental stressors. These include occupational stressors such as low income, protracted working hours and on-call availability^11^. Additionally, veterinarians often possess ready access to lethal drugs, which can be concerning in the context of suicidal ideation^11^. Notably, they are routinely exposed to euthanasia practices in their line of work, an element that holds potential significance for suicidal behaviors^12–14^. Euthanasia is a complex issue that can cause ethical dilemmas, challenging decision-making, moral conflicts, and job-related stress^15–17^. Veterinarians are often placed in situations where they must balance their compassion for animals with the realities of medical and economic constraints, which can lead to emotional exhaustion and moral stress^18^. The complexity is further compounded by veterinarians reporting a deficiency in training regarding decisions pertaining to euthanasia and its administration^17,19,20^. Aside from performing the procedure, veterinarians also have the responsibility of providing assistance and overseeing the feelings of guilt and grief experienced by animal owners^21^. These challenging scenarios, place veterinarians at a higher risk of experiencing significant mental health issues, including suicidal thoughts^12–14^. Indeed, according to a study conducted by Dalum et al. an association was found between the frequency of euthanizing animals and the prevalence of serious suicidal thoughts among these professionals^22^. Specifically, veterinarians who euthanize animals five times or more per week are significantly more likely to experience suicidal ideation^22^.

Also, this practice has the potential to shape one’s perception of death. Specifically, it has been hypothesized that veterinarians may develop an attitude of fearlessness towards death because of their frequent exposure to euthanasia procedures^23^. Substantiating this hypothesis, empirical evidence indicates that veterinarians exhibit a greater willingness to consider euthanasia for humans, in comparison to the general population^24^. Furthermore, studies have demonstrated a significant and positive association between tolerance of suicide (i.e., euthanasia, physician-assisted suicide, and unassisted suicide) and the probability of engaging in suicidal thoughts and behaviors^25,26^. Thus, we hypothesized that the act of euthanasia could be linked to a higher occurrence of suicidal thoughts among veterinarians.

### Individual risk factors

Individual risk factors revolve around intrinsic characteristics and traits. For example, considering demographic characteristics, female veterinarians report more suicidal ideation compared to their male counterparts^22,27^. This trend is consistent with broader findings that suggest females are more likely than males to report suicidal ideation, while males are at a higher risk of completing suicide^28,29^.

Another salient individual risk factor is substance use. The abuse of substances, particularly alcohol, has been robustly linked to an elevated risk of experiencing suicidal thoughts, attempts and deaths^30–32^. Several studies underscore the widespread prevalence of substance and alcohol abuse among healthcare professionals, including veterinarians^33^. A study on drug-related deaths in this occupational group revealed that veterinarians had the highest prevalence of alcohol on postmortem examination^34^. Additionally, veterinarians who use alcohol as a coping mechanism to manage work-related stress were more likely to report suicidal ideation^35^.

Several psychological factors have been examined in veterinarians such as depression, anxiety, and personality traits in relation to suicidal behaviors^36^.

However, there is an underexplored facet of psychological functioning that warrants attention, that is, reflective functioning. Reflective functioning (or mentalization), a concept rooted in attachment theory, is a crucial but overlooked factor to consider in this context^37^. Reflective functioning is defined as the ability to understand and interpret one’s own behaviour, and the behaviour of others, as expressions of mental states (i.e., thoughts, goals, feelings, beliefs, feelings and desires)^38^. Reflective functioning, is linked to several psychological constructs, notably sharing commonalities with empathy and metacognition^39^. Nonetheless, mentalization distinguishes itself by its emphasis on comprehending and identifying mental states. This involves not just interpreting the mental states of others, but also recognizing and reflecting on one’s own internal experiences and emotions.

Hypomentalization is characterized by a limited understanding of one’s own and others’ mental states^40^. When individuals experience challenges in recognizing and comprehending their own mental states, they encounter difficulties in effectively managing their emotions^41^. Hypomentalization can cause difficulties in recognizing and expressing emotions, leading to increased emotional sensitivity or emotional numbing^41^. The absence of emotional understanding and regulation is a crucial element in the formation of different emotional disorders. Indeed, failures in reflective functioning are not only associated with several psychological disorders^42^ and outcomes such as decreased well-being, self-harming behaviors, reduced quality of life, and elevated stress levels; but are currently gaining attention also in relation to suicidality^43–46^. For example, Levi-Belz and Lev-Ari conducted a study on suicide-loss survivors to assess whether difficulties in reflective functioning could explain the link between complicated grief and suicidal thoughts^47^. Importantly, they found that impaired reflective functioning increased the likelihood of suicidal thoughts, with higher levels of failures in reflective functioning being strongly associated with suicidal ideation.

In the context of veterinarians, adequate reflective functioning might play a pivotal role in helping them cope with the emotional challenges they encounter daily, and process emotionally charged events. Impaired reflective functioning, whether excessive or lacking, can cause misinterpretation of behaviors or situations due to an incorrect understanding of one’s own or others’ mental states. However, despite the importance of this psychological factor, no study has been conducted to investigate the role of reflective functioning and its association with suicidal ideation in this vulnerable population.

Thus, this study aims to (i) evaluate the prevalence of veterinarians presenting suicidal ideation, (ii) evaluate the prevalence of veterinarians with a risk of substance abuse, (iii) explore the interplay between sociodemographic (i.e., sex and age), contextual (i.e., exposure to euthanasia) and individual (i.e., substance abuse, reflective functioning) factors and their association with suicidal ideation in a sample of Italian veterinarians. Specifically, regarding the last objective, we hypothesized that difficulties in reflective functioning, greater exposure to animal euthanasia, and increased substance use would result positively associated with suicidal ideation.

## Methods

### Procedure

This study is part of a larger research project on Italian veterinarians’ wellbeing^48^. Ethical approval for this study was obtained by the Research Ethics Board of the University of Parma (Prot. 197561). The research employed a cross-sectional study design, using an anonymous online self-report survey as the primary data collection method. A cohort of licensed veterinarians was recruited as study participants.

To ensure the sample was representative of the broader population of Italian veterinarians, invitations were distributed through the Federation of Italian Professional Veterinary Associations (ANMVI) a national professional association of veterinarians in Italy. In July 2019, an e-mail communication was distributed to a potential cohort of 17,400 participants. The emailed communication outlined the research’s objectives, explicitly identified the ANMVI as the entity overseeing the study and underscored the voluntary and confidential nature of participation.

Data acquisition took place between July 29, 2019, and October 2, 2019. Participants gave their informed written consent through an online platform and completed all survey measures using Survey Monkey. To safeguard participant rights and privacy, it was explicitly conveyed that responses would remain anonymous, and no data that could reveal the identities of the respondents, including their Internet protocol (IP) addresses, were collected.

This study was performed in accordance with the Declaration of Helsinki. Also, the research adhered to ethical standards established by the Ethical Code of the Italian Association of Psychology, the European Code of Conduct for Research Integrity, and the guidelines established by the American Psychological Association.

## Measures

### Predictors

**Demographic characteristics**. The participants filled out a self-reported form that inquired about their age and sex.

#### Contextual factors

Animal euthanasia. Exposure to animal euthanasia was assessed using a single ad hoc item asking respondents how frequently they were exposed to euthanasia cases in a typical month of veterinary practice (ranging from 0 to 30 days).

#### Individual factors

Substance abuse. Substance abuse was evaluated through a specific section of the DSM-5 Self-rated Level 1 Cross-Cutting Symptom Measure—Adult (DSM-XC) questions regarding alcohol and drug abuse, which is composed of three items (i.e., Have you drunk at least four alcoholic beverages in one day?; Have you smoked cigarettes, cigars, or pipe, or did you smoke or chew tobacco?; Have you used it without a medical prescription, in higher doses, or longer than prescribed drugs such as painkillers, stimulants, sedatives, or narcotics?)^49^. The questions referred to the previous two weeks. Scores range from 0 to 4, ranging from “0 = none or not at all” to “4 = severe or nearly every day”, with scores of 1 or higher suggestive of a potential risk of substance abuse. This tool is aligned with the DSM-5 current diagnostic criteria for substance-related disorders, making it comprehensive and appropriate for identifying a broad spectrum of substance abuse issues. Compared to other tools like the Alcohol Use Disorders Identification Test or the Drug Abuse Screening Test, the DSM-XC is more encompassing as it addresses a wider range of substances and behaviors.

Failures in Reflective functioning. Failures in reflective functioning were assessed with the Uncertainty about Mental States (RFQu) subscale from the Reflective functioning questionnaire^50^ that measures hypomentalazing and exhibited a strong and significant association with various clinical attributes, including self-harming behaviors, depressive symptoms, and diminished quality of life^51,52^. The RFQu subscale is composed by 8-items rated on a 7-point Likert scale ranging from “0 = strongly disagree” to “7 = strongly agree”. The focus was on RFQu since The Uncertainty about Mental States (RFQu) subscale includes items such as “Sometimes I do things without really knowing why.” High agreement with RFQu items reflects an inability to understand complex models of one’s own or others’ minds (hypomentalizing), while lower scores suggest an awareness of the complexity and opaqueness of mental states, characteristic of genuine mentalizing. In the present sample, the omega coefficient for the RFQu was 0.70, suggesting a satisfactory internal consistency.

### Outcome

Suicidal ideation. The three questions on suicidal ideation derive from the second National Survey of Psychiatric Morbidity^53^ previously used by Bartram^27^. The questions referred to the previous 12 months (yes/no scoring): “Have you wished that you were dead?”; “Have you felt that life was not worth living?”; “Have you thought of taking your life, even if you would not really do it?”. For determining the prevalence rates, participants scoring 0 on all three questions were categorized as exhibiting an absence of suicidal ideation, while those scoring 1 on at least one question were categorized as exhibiting suicidal ideation. With respect to the structural equation model, a latent variable reflected by the three aforementioned indicators was employed as the study outcome. In the present sample, the omega coefficient calculated at the item level was 0.82, supporting a strong internal consistency.

#### Data analysis

Data were analyzed using IBM SPSS v.25 and Mplus v.8.6.

Descriptive statistics and zero-order correlations were preliminarily calculated. Afterward, the role of difficulties in reflective functioning, exposure to animal euthanasia, and the risk of substance abuse (i.e., predictors) in predicting suicidal ideations (i.e., outcome) was examined using a structural equation modelling approach (SEM). Suicidal ideation was specified as a latent factor using its corresponding three items as manifest indicators, whilst RFQu was defined as a latent dimension reflected by three parcels serving as observed indicators (i.e., aggregates of items;^54^). Parcelling has several advantages over using single indicators, including increased stability in parameter estimates, a higher variable-to-sample size ratio, and a lower likelihood of distributional violations and correlated residuals^55^. Items were assigned to their respective parcels using the *balancing approach* based on the corrected item-total correlations^55^. The goodness of the measurement model was examined using a confirmatory factorial approach (CFA). Composite reliability coefficients were calculated following Fornell and Larcker^56^ for RFQu, whilst for suicidal ideation adjustments were made to account for the dichotomous nature of the items using Green and Yang’s formula^57^. Discriminant validity between the posited factors was examined using the *phi* approach^58^. The *phi* approach entails fixing the estimated correlation parameter between two factors to zero and then conducting a χ2 difference test to compare the constrained and unconstrained models. A significantly lower χ2 value for the unconstrained model indicates that the factors are not perfectly correlated, thus supporting the discriminant validity^59^.

Thereafter, in order to disentangle the unique contributions of the examined factors in explaining the variance of suicidal ideation, we employed a four-step SEM analysis through the saturated correlates approach^60^. The saturated correlates approach demonstrated its superiority in a wide range of empirical conditions for calculating changes in R-squared within a latent variable framework^61^. Specifically, in the first step, demographic confounders including sex (i.e., 0 = males, 1 = females) and age were specified as predictors of suicidal ideation, whilst RFQu, euthanasia and risk substance abuse were specified as saturated correlates. In the second step, the structural path linking euthanasia (i.e., the number of euthanasia cases per month) to suicidal ideation was specified, whilst RFQu and risk of substance abuse were specified as saturated correlates. In the third step, the structural path linking risk of substance abuse (i.e., 0 = not a risk; 1 = at risk) was introduced, whilst RFQu was specified as a saturated correlate. In the fourth step, the direct effect of RFQu on suicidal ideation was included. Among each step, changes in R-squared (ΔR^2^) were calculated and interpreted following Cohen’s conventions^62^: between 0.02 and 0.13 as small, between 0.13 and 0.26 as moderate, and greater than 0.26 as substantial. Due to the dichotomous coding of the suicidal ideation items, they were treated as categorical, and models were estimated using the weighted least squares mean and variance-adjusted estimator (WLSMV)^63,64^.

Following a multifaceted approach^65^, the following criteria were employed to evaluate the fit of the models to the observed data: Tucker and Lewis Index (TLI; values > 0.95 suggest a good fit), comparative fit index (CFI; values > 0.95 suggest a good fit), the root mean square error of approximation (RMSEA; values < 0.05 suggest a close fit), and the standardized root mean square residual (SRMR; values < 0.08 suggest a good fit)^60,61,66,67^. The χ2 likelihood ratio statistic was reported, even considering its sensitivity to detect trivial model misspecifications in large samples^68^.

## Results

### Descriptive statistics and bivariate correlations

Out of the 17,400 solicited participants, 1566 veterinarians responded to the survey, yielding a response rate of 9%. The respondent cohort consisted of 69% males and 31% females, with an average age of 43.86 years (*SD* = 11.32). Most veterinarians, constituting 84.9% of the sample, were employed in companion animal practice. A smaller proportion of the participants, 4.8%, were engaged in the care of production animals, and a minimal percentage of them (i.e., 1.0%), were associated with pharmaceutical or feed companies. Those employed by universities accounted for 1.6% of the sample. Additionally, 3.4% of veterinarians worked within local veterinary units. Lastly, 4.3% of the veterinarians worked in other, unspecified positions.

Among the sample, 44.2% were identified as being at risk of substance abuse, while 29% reported a risk for suicidal ideation. On average, veterinarians were exposed to more than 5 euthanasia cases in a typical month of practice (*M* = 5.43, *SD* = 6.26; range 0–30). Table 1 summarises descriptive statistics and zero-order correlations among the main variables under investigation. As expected, there was a significant positive correlation between RFQu and suicidal ideation (*r* = 0.261, *p* < 0.001). Additionally, the risk of substance abuse was positively associated with suicidal ideation (*r* = 0.130, *p* < 0.001). In terms of demographics, females scored lower on RFQu (*r* = − 0.113, *p* < 0.001), whilst age was negatively associated with risk of substance abuse (*r* = − 0.148, *p* < 0.001) and RFQu (*r* = − 0.123, *p* < 0.001). The other zero-order correlations, although statistically significant due to the large sample size, were found to be negligible in magnitude ( < |.1|)^62^.

**Table 1:** Descriptive statistics and zero-order correlations.

	Mean (SD)/%	1	2	3	4	5	6
1. Sex	69% Males						
2. Age	43.86 (11.32)	0.272***					
3. Euthanasia	5.43 (6.26)	0.032	− 0.043				
4. Risk of substance abuse	44.2% Yes	− 0.003	− 0.148***	0.087**			
5. RFQu	2.83 (3.39)	− 0.113***	− 0.123***	0.058*	0.092***		
6. Suicidal ideation	0.54 (0.96)/29% Yes	− 0.047	− 0.062*	0.065*	0.130***	0.261***	

Sex was codified as 0 = male and 1 = female, and risk of substance abuse was codified as 0 = no and 1 = yes.

*RFQu* Reflective functioning questionnaire—uncertainty. *p < 0.05; **p < 0.01; ***p < 0.001.

### Measurement model

The CFA model positing the two latent dimensions of RFQu and suicidal ideation demonstrated an excellent fit to the observed data: χ2 (8) = 8.373, *p* = 0.397; CFI = 1; TLI = 1; RMSEA = 0.005 (90% CI 0.000–0.030); SRMR = 0.013. More specifically, standardized factor loadings were all statistically significant, ranging from 0.629 to 0.986 (*M* = 0.784; *SD* = 0.134). The composite reliability was acceptable for both RFQu (0.70) and suicidal ideation (0.82). Therefore, we compared the posited CFA with a more parsimonious model in which the estimated correlation parameter between RFQu and suicidal ideation was fixed to unity. The constrained model showed a significant decline in fit (Δχ2 = 263.875, df = 1, *p* < 0.001), supporting the discriminant validity between the two constructs^58^.

### Structural model

**Figure 1:**
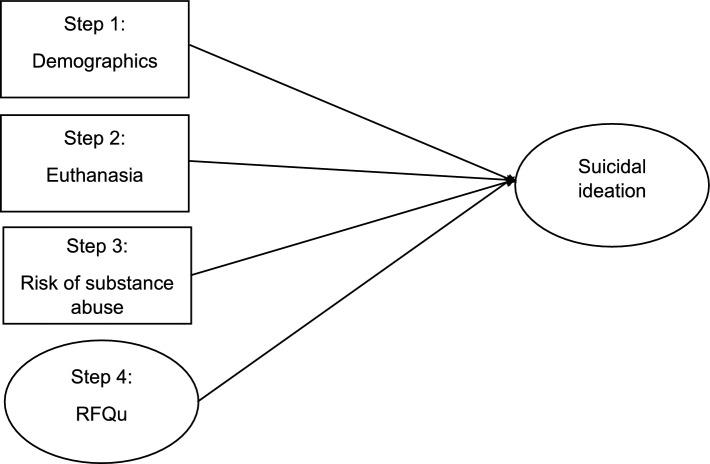
The posited SEM. The four steps were specified through the saturated correlates approach. *Note* Although not represented for clarity, predictors were allowed to correlate.

After establishing the goodness of the measurement model, we proceeded to specify the four-step SEM analysis (see Fig. 1). The four sequentially specified models through the saturated correlates approach had the same degrees of freedom and exhibited an almost perfect fit to the observed data: χ2 (24) = 31.743, *p* = 0.133; CFI = 0.998; TLI = 0.997; RMSEA = 0.014 (90% CI 0.000–0.027); SRMR = 0.017.

In the first step, sex (B = − 0.091, *p* = 0.205) and age (β = − 0.069, *p* = 0.056) were not significantly associated with suicidal ideation. Consequently, demographic variables explained a negligible amount of variance in the outcome (R^2^ = 0.009).

In the second step, we observed a significant association between euthanasia and suicidal ideation (β = 0.084, *p* = 0.014). However, the inclusion of this structural path resulted in a negligible incremental amount of variance (ΔR^2^ = 0.007).

In the third step, the structural path linking the risk of substance abuse to suicidal ideation led to a small incremental amount of variance (ΔR^2^ = 0.025). Specifically, the risk of substance abuse was positively associated with suicidal ideation (B = 0.285, *p* < 0.001).

In the fourth step, the inclusion of the structural path linking RFQu to suicidal ideation contributed to a small-to-moderate incremental amount of variance (ΔR^2^ = 0.114). In detail, RFQu was positively associated with suicidal ideation (β = 0.345, *p* < 0.001). Importantly, even after including RFQu in the equation, the unique contribution of the risk of substance abuse remained statistically significant (B = 0.232, *p* < 0.001). In sum, the entire model accounted for approximately 16% of the variance of suicidal ideation (see Table 2). According to Ferguson^69^, the magnitude of the R-squared can be interpreted as practically significant for social science data.

**Table 2:** Four-step SEM analysis through the saturated correlates approach.

	Step 1	Step 2	Step 3	Step 4
Predictor	Suicidal Ideation	Suicidal ideation	Suicidal ideation	Suicidal ideation
Sex	B = − 0.091	B = − 0.099	B = − 0.110	B = − 0.038
Age	β = − 0.069	β = − 0.064	β = − 0.039	β = − 0.005
Euthanasia		β = 0.084*	β = 0.071*	β = 0.050
Risk of substance abuse			B = 0.285***	B = 0.232***
RFQu				β = 0.345***
R^2^	0.009	0.016	0.041	0.155
ΔR^2^		0.007	0.025	0.114

All coefficients are reported in a completely standardized metric (β), except for sex (coded as 0 = female and 1 = male) and risk of substance abuse (coded as 0 = no and 1 = yes) which are reported in their unstandardized form (*B*). RFQu: Reflective Functioning questionnaire—uncertainty.

* *p* < .05; ** *p* < .01; *** *p* < .001.

## Discussion

The present study aimed to evaluate the prevalence of suicidal ideation, the risk of substance abuse, and the interplay of sociodemographic, contextual, and individual factors in a sample of Italian veterinarians.

### Prevalence of substance abuse risk and suicidal ideation

The finding that 44.2% of veterinarians exhibit a potential risk for substance abuse is a matter of significant concern within the context of mental health and well-being, particularly in a profession that demands a high degree of responsibility and empathy. This finding is partially consistent with previous evidence. For example, in Bartram’s study, the risk of alcohol-related issues was evidenced in 62.6% of the sample^63^. It is important to acknowledge that the discrepancy may be attributable to methodological differences in the evaluation of substance-related risks. In our study, we examined a broader spectrum of substance abuse risk, whereas Bartram’s study focused primarily on alcohol consumption. In addition, the differences in assessment tools play a crucial role in the observed differences. We used the DSM XC whereas Bartram and colleagues used the Alcohol Use Disorders Identification Test. For context, data from a 2017 general population survey on drug use in Italy revealed that about one-third of the Italian population aged 15–64 had experimented with psychoactive substances at least once in their lifetime^70^. Moreover, 10% of the population reported using these substances within the past year. A comprehensive survey conducted in 2017 gathered data from 101,002 telephone interviews with Italian residents aged 18–69. This extensive study found that 6.5% of respondents engaged in unhealthy alcohol^71^. However, it is important to note that the methodologies used to assess substance use in the general population and among veterinarians differ significantly. As a result, direct comparisons of these percentages should be approached with caution. The differing instruments and methods of data collection can lead to variances in reported prevalence rates, making it challenging to draw direct comparisons between the general public and specific professional groups like veterinarians.

In this context, alcohol use can be conceptualized as a dysfunctional coping modality. Indeed, veterinarians often confront emotionally taxing situations, such as dealing with the suffering and euthanasia of animals, long working hours, and the pressure to uphold the health of both animals and their human owners^8^. These stressors can contribute to an increased vulnerability to substance abuse as a coping mechanism. Accordingly, the findings of a previous study examining drinking motives among veterinarians reveal that veterinary students who consume alcohol as a means to manage their internal state and pursue gratification are more prone to engaging in risky drinking behaviors^72^. Therefore, the observed high prevalence of risk in both studies, suggesting that about one in two veterinarians are at risk for substance abuse, deserves clinical attention, especially in terms of education and prevention.

Moreover, the fact that 29% of the veterinarian sample exhibited suicidal ideation is a matter of significant concern from a clinical and epidemiological perspective. This result is notably higher than the 19.2% prevalence of suicidal ideation in the past 12 months reported in Schwerdtfeger’s study among veterinarians in Germany^65,73^. Multiple factors, including economic and sociocultural differences, healthcare systems, and societal attitudes toward mental health, may contribute to these disparities. Not least, the evaluation methods for suicidal ideation may also influence the results. Also, the rates of suicidal ideation within the veterinary community are considerably higher when compared to the estimated lifetime prevalence of 3% within the Italian general population^74^. Therefore, our study suggests that a need exists to support the mental health of veterinarians and foster their psychological well-being.

### Demographic, contextual and individual predictors of suicidal ideation

SEM results suggest that demographic factors such as sex and age are not significantly associated with suicidal ideation in the veterinarian population, while contextual and individual factors emerged as significant predictors.

Euthanasia, a significant and emotionally charged aspect of veterinary practice, has long been recognized as a major source of stress for veterinarians. In our sample, the mean number of euthanasia performed by veterinarians in a month was five. This result aligns with another study conducted on Austrian veterinarians (*N* = 486) working in small animal practices, which reported an average of three euthanasia per month. However, our results suggest that the impact of euthanasia on suicidal ideation may not be as pronounced as initially expected. In fact, in the final model euthanasia emerged as a non-significant predictor of suicidal ideation. In another study conducted by Dalum et al. on 2350 veterinarians, it was reported that performing five or more euthanasia per week is associated with the presence of suicidal ideation in the past year^22^. Therefore, it can be hypothesized that it is not merely the exposure to euthanasia, but also the frequency with which it is conducted, that may have an impact on suicidal ideation.

Veterinarians witness the emotional aftermath of mortality, both in terms of its impact on animals and the emotional burden it places on clients. It has been suggested that due to their role in guiding clients through end-of-life decisions, veterinarians may be exposed to clients’ grief and suffering, which might serve as a reminder of the pain their loved ones might face in the event of their suicide^75^.

Also, of particular interest is the concept of emotional transference. It might be argued that veterinarians may experience a unique form of emotional buffering through repeated exposure to clients’ expressions of gratitude and positive emotions following euthanasia procedures^76,77^. Emotional transference may act as a protective mechanism, mitigating the negative psychological impact of euthanasia. This suggests that positive feedback and emotional support may counterbalance the burden of euthanasia.

Importantly, substance abuse emerged as a significant predictor of suicidal ideation risk, in line with evidence regarding other populations^78,79^. For example, the relationship between alcohol abuse and suicidal behavior is well established, and alcoholism has long been recognized as a major contributor^80,81^. However, in the context of veterinary profession evidence are scant. To the best of our knowledge, only one study by Bartram and colleagues investigates alcohol consumption and its association with mental health outcomes, including suicidal ideation among vets in the UK. However, in contrast with our findings, they found that the level of alcohol consumption did not appear to be associated with the 12-month prevalence of suicidal ideation^82^.

Furthermore, difficulties in reflective functioning were positively associated with the presence of suicidal ideation in our study, aligning with previous evidence on other populations. For example, reflective functioning was independently associated with higher suicide risk in psychiatric inpatients^83^. Additionally, a study involving 152 suicide-loss survivors demonstrated a positive association between suicidal ideation and difficulties in reflective functioning. Our results are in line with the theoretical framework of reflective functioning. Individuals with adequate reflective functioning capacity exhibit resilience in stressful situations^84^. Notably, this capacity enables individuals to explore and verbalize challenging experiences, facilitating the ability to seek and receive help^85^. Conversely, those with reflective functioning difficulties encounter challenges in accessing explicit-controlled reflective functioning, particularly when faced with heightened emotional arousal or stress. Reflective functioning, acting as an affective-cognitive mechanism, allows individuals to consider diverse perspectives and comprehend the underlying reasons for their behavior, playing a crucial role in emotion regulation. Accordingly, deficits in reflective functioning may lead to maladaptive emotion regulation strategies, posing challenges in recognizing and managing emotions^86^. This, in turn, can result in social isolation, aggressive behaviors, and the development of various psychological symptoms, including suicidal ideation.

Several limitations should be considered. The cross-sectional design impedes causal inferences; however, it provides valuable associations that can inform longitudinal studies aimed at exploring these relationships over time. Moreover, the euthanasia measure in our study assessed the frequency of exposure in the past 30 days; future studies might benefit from evaluating both recent and lifetime cumulative euthanasia exposure to capture a broader impact. Furthermore, we acknowledge the temporal discrepancy between assessing suicidal ideation over the past 12 months and other variables over shorter periods. Future research should aim for consistency in time frames to enhance the predictive contribution. Another limitation of this study is the potential for non-response bias. Indeed, we achieved a response rate of 9%, it is possible that veterinarians experiencing higher levels of stress or suicidal ideation were more likely to participate, potentially biasing the results. Future research should aim to address this by employing strategies to increase response rates and assess the characteristics of non-respondents.

Notwithstanding its limitations, the findings of this study carry significant implications. Firstly, substance abuse prevention and intervention programs are imperative for reducing the risk of suicidal ideation. Secondly, mental health interventions for veterinarians should prioritize reducing difficulties with reflective functioning, as these difficulties appear to be a critical factor associated with the presence of suicidal ideation. Notably, mentalization-based treatment has been found to significantly reduce self-harm and parasuicidal behaviors in patients with borderline personality disorder, and evidence on adolescents suggests that positive changes in reflective functioning serve as mediating factors in reducing self-harm^87,88^. These findings hold significant clinical importance, as they indicate that preventive interventions informed by a mentalization-based approach can be tailored specifically for this particular group.

## Data Availability

The data that support the findings of this study are available from the corresponding author but restrictions apply to the availability of these data, which were used under license for the current study, and so are not publicly available. Data are however available from the authors upon reasonable request.
