# Author Correction: Predictors of suicidal ideation in Italian veterinarians

**DOI:** 10.1038/s41598-024-71391-6

**Published:** 2024-09-05

**Authors:** Giorgia Varallo, Andrea Zagaria, Valentina Baldini, Alessandro Schianchi, Marta Brscic, Matteo Panero, Christian Franceschini, Adriano Schimmenti, Alessandro Musetti

**Affiliations:** 1 Department of Biomedical, Metabolic and Neural Sciences, University of Modena and Reggio Emilia, Modena, Italy; 2 Department of Psychology, Sapienza University of Rome, Rome, Italy; 3 Department of Biomedical and Neuromotor Sciences, University of Bologna, Bologna, Italy; 4 Fornovo di Taro, Parma, Italy; 5 Department of Animal Medicine, Production and Health (MAPS), University of Padova, Viale dell’Università 16, 35020 Legnaro, PD Italy; 6 Department of Neuroscience “Rita Levi Montalcini”, Eating Disorders Center, University of Turin, Turin, Italy; 7 Department of Medicine and Surgery, University of Parma, Parma, Italy; 8 Faculty of Human and Social Sciences, UKE - Kore University of Enna, Enna, Italy; 9 Department of Humanities, Social Sciences and Cultural Industries, University of Parma, Parma, Italy

Correction to: *Scientific Reports* 10.1038/s41598-024-68330-w, published online 30 July 2024

The original version of this Article contained an error in the Methods section where the below Reference was omitted, which is listed as Reference 48.

48. Musetti, A., Schianchi, A., Caricati, L., Manari, T., & Schimmenti, A. (2020). Exposure to animal suffering, adult attachment styles, and professional quality of life in a sample of Italian veterinarians. *PloS one*, *15*(8), e0237991.

As a result, in the Methods section, under the subheader ‘Procedure’,

“This study is part of a larger research project on Italian veterinarians’ wellbeing (BLINDED FOR REVIEW PURPOSES).”

now reads:

“This study is part of a larger research project on Italian veterinarians’ wellbeing (48).”

As a result of the changes, the References have been renumbered.

The original Article has been corrected.

